# Transverse Presentation

**Published:** 1889-07

**Authors:** Clarence Willard Butler

**Affiliations:** I. H. A., Bureau of Obstetrics


### TRANSVERSE PRESENTATION; A CASE WITH REMARKS.

CLARENCE WILLARD BUTLER, M. D.

[I. H. A., Bureau of Obstetrics.]

At ten o'clock P. M., September 15th, 1887, I was called by telephone to see Mrs J. The message did not state the nature of the trouble for which Mrs. J. desired medical attention, and not knowing that she was, as the Germans say, "of good hopes," I found myself on my arrival at her bedside in attendance upon a case of labor, three miles from my office and with a pocket medicine-case as my sole armamentarium.

Mrs. J. was about thirty years of age, the wife of a mechanic in straitened circumstances, the mother of two living children, and has suffered from, and, as I afterward learned, was the procurer of four abortions. The means which she employed for this nefarious purpose she would not divulge. Since the first month after her marriage, ten years previous, she had been pregnant or nursing almost without intermission. Physically she was short, stout, and fat, especially about the abdomen.

Upon examination the os uteri was found dilated to about the size of the traditional quarter of a dollar. No presenting part of the fœtus was to be felt. Fearing an abnormal position, abdominal examination was made, but on account of the extreme adiposity of the abdominal parietes a diagnosis was impossible to me. Determining to wait for wider dilatation, I instructed the nurse to call me in two hours' time, and went to bed in an adjoining room. You have all in your practice met the old woman who approaches a "laying-out" or a "lying-in" with the same morbid delight, and who in the latter class of cases beguiles the tedious hours of parturition with circumstantial accounts of all the cases of difficult and disastrous labor which have occurred within her wide experience and observation, duly embellished by her vivid imagination. Possessed of unbounded confidence in her own skill and knowledge in all matters tocological, the suggestions of the medical attendant she superciliously ignores, and his most explicit directions she willfully disobeys. Both advice and medicine, unless closely watched, she does not hesitate to administer, and it is impossible to tell which is the more baneful of the two. In short, she is the lineal descendant of the ancient witches, and chiefly renowned, like her more celebrated ancestors, for her pernicious activity in raising the Devil.

Such an uncomfortable old woman was the nurse and relative as well of my patient. Not trusting to her discretion, I did not inform her of my fears, while she, satisfied in her great wisdom that all was progressing favorably, did not call me until seven o'clock the next morning, and then only because the waters had broken nearly an hour before, and, although the pains were regular and quite severe, they were not expulsive. An examination now revealed a transverse position (right-illiac, dorso-abdominal), the side presenting at the mouth of the womb, with a point about corresponding with the angle of the scapula, over the centre of the orifice. The os being now widely dilated, podalic version was determined upon, but, upon attempting to introduce the hand, so irritable was the os and so violent its contractions, that after repeated attempts I desisted, deeming such manipulation even dangerous without anæsthesia. Accordingly, I determined to go at once to my office for the anæsthetic. Although this necessitated an absence of more than an hour, I did not apprehend serious results from the delay because of the excellent general condition of the patient. At a quarter to eight A. M. I dropped a dose of Pulsat.$^{mm}$ (Tafel) upon her tongue and departed.

At nine o'clock on my return I found the head and shoulders of the child already born, the nates and legs being still within the vagina.

The head had been delivered with the occiput toward the symphysis pubis. The infant, which was dead, weighed six and one-half pounds. Mrs. J. reported that the pains became more severe after my departure though less frequent, and in due time the head was born. Nothing else worthy of mention occurred.

She made a good recovery without complications.

There are some points connected with this case which seem to me worthy of consideration, and I shall refer to them. In the first place, I am satisfied that this was a case of criminal abortion, in spite of the protestations of the patient that such was not the case; indeed, this opinion was confirmed by the manner and vehemence of the protestations.

"The lady doth protest too much, methinks."

The sensitiveness of the os uteri and the sharp and sudden contraction of the circular fibres at this part on touch, with the death of the child, make it probable that ergot of rye was the drug used for the purpose. Further examination of the preparturient symptoms revealed the fact that motion, which had been growing weaker for some days, had ceased entirely nearly a week before the commencement of labor. Indeed, there seems little doubt that fœtal life had ceased some days before the birth, and this would favor the opinion held by most writers that viability is an important factor in the determination of the child's position within the uterine cavity.

There could have been no error in diagnosis in the case. The os was well dilated, the amniotic fluid had escaped, and there was nothing which could interfere in any degree with the easy recognition of the presenting part and its relations to the os, except the violent contractions of the unduly irritable uterus—a difficulty so insignificant and easily avoided that the veriest neophyte could hardly have blundered. Were it not true that there seems a disposition on the part of certain prejudiced critics to carp at all cases as insignificant, or to question the diagnosis where favorable remedial action is claimed for highly potentized drugs, the foregoing remarks would be unnecessary, but in view of such fact, an expression of the writer's confidence in his diagnosis may not be superfluous.

Here, then, was a case of trunk presentation converted by version into a cephalic and the second stage of labor completed in about an hour. Was this version spontaneous, or was it a direct result of the action of a single dose of a very high potency of Pulsatilla?

The case is unprecedented so far as I have been able to discover, and since no positive knowledge, perhaps no approximate estimate of the value of a drug in any condition can be arrived at from one case, only by further observation can this point be decided. It is not likely that such observation will occur, for, in the first place, the presentation of the trunk may be expected in only one case out of every two hundred and thirty-one according to Swayne [*Obstetric Aphorisms*] while Velpeau, in tables collected from many sources, finds that in fifty-four thousand seven hundred and twenty-three cases the trunk presented on an average once in two hundred and thirty-four times, and in forty-eight thousand one hundred and sixty cases cited by Ramsbotham the trunk presented one hundred and fifty-eight times, or about once in three hundred and fourteen labors. Of these cases, the great majority were undoubtedly shoulder presentations or quickly became such, as experience amply shows, in which the probabilities of version are much less, since the shoulder from its salient form will usually be arrested at the superior straight, a position which favors spontaneous evolution, but not version. The proportion of cases then favorable to spontaneous version is a very small one, and from these must be deducted the large majority, since it will very seldom occur that accident will prevent the accoucher from performing podalic-version, which is his plain and undoubted duty. The settlement of the question of Pulsatilla's possible action then may be considered as practically impossible by repeated experiences. But I do not hesitate to avow my own belief in its efficacy, and my reasons for such belief will be given for your consideration. Let us, if you please, consider the natural processes necessary to spontaneous version. The muscular fibres of the uterus are arranged in three groups, the so-called circular fibres, but which are circular only at the lower part of the body of the uterus and at the os; they embrace the larger part of the cavity of the uterus at "low angles of intersection" [Allen's *Human Anatomy*, sec. 6, article Uterus]; the variously placed oblique bands, and the longitudinal fibres which, situated most largely on the posterior surface in the unimpregnated, become in the gravid uterus an important factor from the extent and amount of their development. The action of all of these muscles throughout the upper body and fundus of the uterus when provoked in labor to action, after the os is dilated, is to narrow the cavity of the uterus in all directions except from below upward. From the arrangement of these fibres, and from their greater development in the upper body and the fundus of the uterus, the pressure upon the uterine contents must be greater from the fundus toward the os.

The circular fibres intersecting at low angles, bring pressure downward though indirectly; the oblique bands, also, though much more powerfully, indirectly exert pressure in the same direction, while the longitudinal muscular fibres almost directly narrow the uterine cavity in its longitudinal axis.

During labor these muscles bring the uterus closely to that part of the fœtus most nearly in contact with them. This "moulding" of the uterus to the contents is in accord with the laws of muscular action elsewhere, viz.: that muscular fibre is shortened under action in the line of its long axis.

If now for some unknown cause the long axis of the fœtus has not, as it naturally should, accommodated itself to the long axis of the uterine cavity, and instead of a breach or a head presenting, the position is transverse, what would be the natural action of the uterus in its endeavor to void its unwelcome contents? It can act but one way. Wherever a part of the child comes closely enough in contact with the muscular fibres to provoke them to activity, this muscular activity brings the uterus closely against that part, moulding it to it as if the position was a normal one, and since the greatest pressure would be exerted upon that part which from its size or position irritates to such natural action the greatest amount of muscular fibre, the tendency would be to press it toward the os as the point of least resistance, and, as well because the aggregate of uterine muscular pressure is as already stated, in this direction.

That part, then, will be subjected to the greatest amount of this muscular pressure which, from the position of the child, is highest in the uterine cavity, because it presents the greatest surface to muscular action and because it presents this surface to that portion of the uterus which is most muscularly developed. The natural tendency, therefore, would be by pressing either the head or breach as might be, toward the os uteri, to convert a transverse into a longitudinal position. The difficulties in accomplishing this are several and great, and it is no wonder that Richardson says [*Obstetrics*, 1887, p. 33] "it is certain that change of presentation after the escape of the waters is scarcely possible."

Of those difficulties the first and most important is the contra pressure brought to bear upon the extremity of the child which presents itself at the other side of the uterine cavity. The usual explanation, indeed the only one I have found, is that, while the muscles on the one side contract powerfully, those on the other relax, thus allowing the descent of the one extremity and the ascent of the other. I do not see how any such theory is tenable or can be entertained even for a moment. I know of no physiological law by which muscular fibre, under irritation from pressure, relaxes. There is none, and in all cases where version is commenced in one direction it is carried on by the superior force imparted by the muscles of the one side and in spite of antagonistic muscular action on the other. The uterus, however, having moulded itself to one part, in some measure modifies this antagonistic action by changing the uterine outline, and accordingly the direction of the muscular fibres, so that there is less direct downward and antagonistic pressure upon the less active side. Indeed, it is probable that the lateral pressure exerted by this direction of muscular fibre may be in some slight measure auxiliary to the process already begun, although it must be slight as compared with the inimical action. It would seem that after a certain portion of the labor necessary to produce the descent of that part originally subjected to the greatest pressure, and, therefore, under that pressure carried toward the natural outlet, had been accomplished between the forces exerted by the two sides of the uterus, that further progress would be impossible. This does not occur, however, principally I am of opinion because of the original moulding of the uterus, because of the necessary change of the direction of the muscular fibres of the opposite side by this process; and because of the well-known fact that a muscle under continued stimulation to certain work becomes more irritable, and consequently more prompt in its action under the same stimulus up to the point of temporary paresis from exhaustion. Another difficulty to be overcome, and often one not possible to be overcome by nature's unaided efforts, is the arrest of the shoulder at the superior straight, but this accident, if it occur, introduces considerations wholly foreign to the subject now under consideration. I think, therefore, that it may be safely assumed that in all cases of transverse position the effort of nature unaided is to convert this transverse into a longitudinal position; in short, that the *attempt* of nature is always to accomplish spontaneous version. If this, then, is the natural process, and it seems to be, is it a matter of wonder that a remedy may have such effect upon the system as to further that process? No one thing in drug action is more certain than that drugs have the power, by their inherent affinity for certain tissues and organs, to modify their functions, and even to change their structure, and no one thing in remedial action is more positive than that certain remedies have a marked power in controlling and regulating muscular action—especially perhaps uterine muscular action. Of all drugs which have the power to change irregular and inefficient labor-pains into regular and efficient ones, none is better known, none more often needed, (indicated by definite symptoms, of course) than Pulsatilla. This is a fact so well known that no proof need be offered. If, then, the process accomplished in the case recorded was a natural one; if the drug used has an undoubted and acknowledged power over the natural processes of labor; if the version was accomplished more rapidly and more easily than any other on record, why should we doubt that the efficient factor in the favorable result was the action of the remedy?

